# KCNQ and KCNE Isoform-Dependent Pharmacology Rationalizes Native American Dual Use of Specific Plants as Both Analgesics and Gastrointestinal Therapeutics

**DOI:** 10.3389/fphys.2021.777057

**Published:** 2021-11-11

**Authors:** Geoffrey W. Abbott, Kaitlyn E. Redford, Ryan F. Yoshimura, Rían W. Manville, Luiz Moreira, Kevin Tran, Grey Arena, Alexandra Kookootsedes, Emma Lasky, Elliot Gunnison

**Affiliations:** ^1^Bioelectricity Laboratory, Department of Physiology and Biophysics, School of Medicine, University of California, Irvine, Irvine, CA, United States; ^2^Redwood Creek Vegetation Team, National Park Service, Sausalito, CA, United States

**Keywords:** KCNQ channels, KCNE subunits, analgesia, botanical medicines, Native American

## Abstract

Indigenous peoples of the Americas are proficient in botanical medicine. KCNQ family voltage-gated potassium (Kv) channels are sensitive to a variety of ligands, including plant metabolites. Here, we screened methanolic extracts prepared from 40 Californian coastal redwood forest plants for effects on Kv current and membrane potential in *Xenopus* oocytes heterologously expressing KCNQ2/3, which regulates excitability of neurons, including those that sense pain. Extracts from 9 of the 40 plant species increased KCNQ2/3 current at –60 mV by ≥threefold (maximally, 15-fold by *Urtica dioica*) and/or hyperpolarized membrane potential by ≥-3 mV (maximally, –11 mV by *Arctostaphylos glandulosa*). All nine plants have traditionally been used as both analgesics and gastrointestinal therapeutics. Of two extracts tested, both acted as KCNQ-dependent analgesics in mice. KCNQ2/3 activation at physiologically relevant, subthreshold membrane potentials by tannic acid, gallic acid and quercetin provided molecular correlates for analgesic action of several of the plants. While tannic acid also activated KCNQ1 and KCNQ1-KCNE1 at hyperpolarized, negative membrane potentials, it inhibited KCNQ1-KCNE3 at both negative and positive membrane potentials, mechanistically rationalizing historical use of tannic acid-containing plants as gastrointestinal therapeutics. KCNE dependence of KCNQ channel modulation by plant metabolites therefore provides a molecular mechanistic basis for Native American use of specific plants as both analgesics and gastrointestinal aids.

## Introduction

The use of plants as medicines predates human history (Inskeep, 1969; Hardy et al., 2012; Weyrich et al., 2017). Indigenous peoples of North America have used thousands of plant species as medicines (Moerman, 2009). The Ohlone and Coast Miwok peoples inhabited for more than 10,000 years land in what is now Marin County, including dense redwood forest in the Redwood Creek watershed. The Coast Miwok managed the various environments in this area, from beach to grassland to forest, and were adept at utilization of plants for a variety of purposes, including as food and medicine (Heizer and Whipple, 1971; Bean, 1994).

**FIGURE 1:**
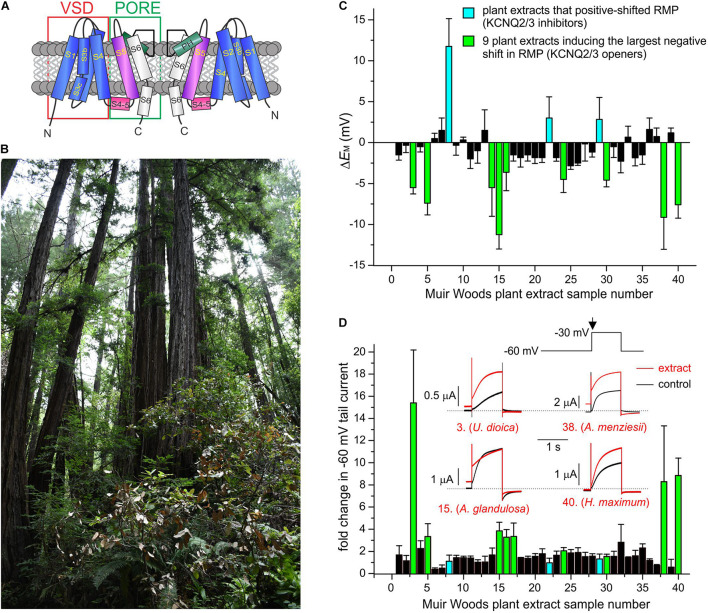
Screening Muir Woods plant extracts for KCNQ2/3 opening activity. All error bars indicate SEM. *n* = number of oocytes. **(A)** Topological representation of a Kv channel showing two of the four subunits that comprise a channel. PH, pore helix. VSD, voltage sensing domain. **(B)** Image of area of Muir Woods from which plants were collected. **(C)** Screen of 40 Muir Woods plant extracts (1:50 dilution) showing their effects on *E*_M_ (Δ*E*_M_ compared to control) of *Xenopus* oocytes expressing human KCNQ2/3; *n* = 3–11 per extract. **(D)** Screen of 40 Muir Woods plant extracts (1:50 dilution) showing their effects on *Xenopus* oocytes-expressed KCNQ2/3 current at –60 mV (fold-change compared to control) *n* = 3–8 per extract. Columns colored according to key in panel **(C)**. Inset: exemplar KCNQ2/3 tail current traces in bath solution (black) or with each of four of the KCNQ2/3-activating plants extracts in the screen (red), using the voltage protocol shown upper right. Arrow: time point on voltage protocol at which tail currents are measured. Dashed line here and throughout indicates zero current level.

In many cases, the mechanisms underlying the purported beneficial effects of traditional botanical medicines are unknown. Voltage-gated potassium (Kv) channels (Figure 1A) within the KCNQ (Kv7) subfamily were recently recognized as important targets for secondary metabolites found in medicinal plants used by indigenous peoples in areas including Africa, Asia, the Caribbean, and Latin America (Matschke et al., 2016; Manville and Abbott, 2018, 2019; Manville et al., 2019).

Heteromeric KCNQ2/3 channels are considered the primary molecular correlate of the neuronal M-current, a subthreshold-activating Kv current that regulates neuronal excitability, with additional contribution from KCNQ3/5 and possibly homomeric KCNQ2, KCNQ3, KCNQ5, and other heteromeric KCNQ channels (Biervert et al., 1998; Singh et al., 1998; Wang et al., 1998; Tzingounis et al., 2010; Klinger et al., 2011). KCNQ2/3 channels are also expressed in nociceptive neurons and are candidate targets for pain medications (Du et al., 2018).

As well as their capacity to detect and activate in response to voltage changes across the cell membrane, KCNQ channels can also bind and open in response to small molecules, endowing them with some ligand-gating properties in addition to their primary classification as voltage-gated (Abbott, 2020). Direct binding of the anticonvulsant, retigabine, shifts the voltage dependence of KCNQ2/3 activation such that it can open at more hyperpolarized potentials, a property that diminishes seizure activity by disfavoring hyperexcitability (Main et al., 2000; Wickenden et al., 2000). A similar effect may underlie the pain-relieving effects of retigabine (Blackburn-Munro and Jensen, 2003; Abd-Elsayed et al., 2015; Abd-Elsayed et al., 2019). The neurotransmitter GABA can also bind to KCNQ3 and KCNQ5 subunits, in a similar binding pocket to that of retigabine (Schenzer et al., 2005; Lange et al., 2009), suggesting an evolutionary basis for ligand sensitivity of KCNQ channels *via* the retigabine binding pocket (Manville et al., 2018; Manville and Abbott, 2020). GABA binding also increases channel activity at subthreshold potentials, reducing cellular excitability (Manville et al., 2018). Furthermore, a variety of plant secondary metabolites can each occupy the retigabine/GABA binding pocket, an effect that provides a mechanistic basis for the anticonvulsant and vasorelaxant effects of some medicinal plants (Matschke et al., 2016; Manville and Abbott, 2018, 2019; Manville et al., 2019).

Here, we used electrophysiological assays to screen for KCNQ2/3 activating properties a random sample of 40 plant extracts from Californian coastal redwood forest. We demonstrate a striking link between KCNQ isoform-specific modulatory activity and historical usage as both folk medicine analgesics and gastrointestinal aids, confirm efficacy and KCNQ dependence in a mouse pain model, and identify effects and their molecular mechanisms of constituent compounds tannic and gallic acid on KCNQ channels, as molecular correlates of the plants’ analgesic and gastrointestinal effects.

## Materials and Methods

### Plant Collection and Preparation of Plant Extracts

Plant samples comprising aerial portions (primarily leaves) were collected July 1–3, 2019 from Muir Woods National Monument under permit # MUWO-2019-SCI-0003, refrigerated for several days during the collection period and then frozen until the day of extraction. We homogenized samples using a bead mill with porcelain beads in batches in 50 ml tubes (Omni International, Kennesaw, GA, United States). We then performed methanolic extractions (80% methanol/20% water) on the homogenates for 48 h at room temperature, with occasional inversion of the bottles to resuspend the extracts. The extracts were then filtered through Whatman filter paper #1 (Whatman, Maidstone, United Kingdom), and then the methanol was removed by evaporation in a fume hood for 24–48 h at room temperature. We next centrifuged extracts for 10 min at 15°C, 4000 RCF to remove the remaining particulate matter, followed by storage at –20°C. On the day of electrophysiological recording, we thawed the extracts and diluted them 1:50 in bath solution (see below) immediately before use.

### Channel Subunit cRNA Preparation and *Xenopus laevis* Oocyte Injection

As previously described (Manville and Abbott, 2018), we generated cRNA transcripts encoding human KCNQ1, KCNQ2, KCNQ3, KCNE1, and KCNE3 by *in vitro* transcription using the mMessage mMachine kit (Thermo Fisher Scientific), after vector linearization, from cDNA sub-cloned into plasmids incorporating *Xenopus laevis* β-globin 5′ and 3′ UTRs flanking the coding region to enhance translation and cRNA stability. We injected defolliculated stage V and VI *X. laevis* oocytes (Xenoocyte, Dexter, MI, United States) with KCNE (2 ng) and KCNQ cRNAs (10 ng, KCNQ1; 1.5–5 ng, wild-type and mutant KCNQ2/3). We incubated the oocytes at 16°C in ND96 oocyte storage solution containing penicillin and streptomycin, with daily washing, for 2–3 days prior to two-electrode voltage-clamp (TEVC) recording.

### Two-Electrode Voltage Clamp

We performed TEVC at room temperature using an OC-725C amplifier (Warner Instruments, Hamden, CT) and pClamp10 software (Molecular Devices, Sunnyvale, CA) 2–3 days after cRNA injection as described in the section above. For recording, oocytes were placed in a small-volume oocyte bath (Warner Instruments) and viewed with a dissection microscope. We sourced chemicals from Sigma-Aldrich, St. Louis, MO. We studied effects of plant extracts and of compounds previously identified in *Arbutus, Arctostaphylos, Polystichum, and Urtica* sp. leaf extracts, solubilized directly in bath solution (in mM): 96 NaCl, 4 KCl, 1 MgCl_2_, 1 CaCl_2,_ 10 HEPES (pH 7.6). We introduced extracts or compounds into the oocyte recording bath by gravity perfusion at a constant flow of 1 ml per minute for 3 min prior to recording. Pipettes were of 1–2 MΩ resistance when filled with 3 M KCl. We recorded currents in response to voltage pulses between –80 and +40 mV at either 10 or 20 mV intervals from a holding potential of –80 mV, to yield current-voltage relationships and examine activation kinetics. We analyzed data using Clampfit (Molecular Devices) and Graphpad Prism software (GraphPad, San Diego, CA, United States), stating values as mean ± SEM. We plotted raw or normalized tail currents versus prepulse voltage and fitted the points with a single Boltzmann function:


(1) $$g=\frac{\left(A_{1}-A_{2}\right)}{\left\{1+e\,x\,p\,\left[V_{\frac{1}{2}}-V/V\,s\right]\right\}\,y+A_{2}}$$

where *g* is the normalized tail conductance, A_1_ is the initial value at –∞, A_2_ is the final value at +∞, V_1/2_ is the half-maximal voltage of activation, and V_s_ the slope factor.

### Cell Culture and Transfections

Chinese Hamster ovary (CHO) cells were grown in cell culture flasks in DMEM with 10% fetal bovine serum and 1 % penicillin and streptomycin in a humidified incubator at 37°C (5% CO_2_). Cells were passaged every 3 days and discarded after ∼30 passages. For transfection, we used TrasnIT-LT1 (Mirus Bio LLC, Madison, WI, United States) with Opti-MEM Reduced Serum Medium. CHO cells were plated on 24 well plates on plastic coverslips and transfected 24 h later using 0.5 μg each of human KCNQ2, human KCNQ3, and eGFP cDNA (to identify transfected cells).

### Whole-Cell Patch Clamp

Patch-clamp electrophysiology experiments were performed as before (Manville et al., 2018; Harkcom et al., 2019; Tedeschi et al., 2021) on CHO cells at room temperature (22–25°C). Using a whole-cell patch configuration under voltage-clamp conditions, data were obtained with an Axopatch Multiclamp 700A apparatus digitized and analyzed using pClamp 9.2 (Axon Instruments, Forster City, CA), together with Graphpad Prism 9 (Graphpad; La Jolla, CA, United States). The pipettes were pulled from borosilicate glass capillaries (World precision Instruments) using a P-97 micropipette puller (Sutter Instruments, Novato, CA) and had a resistance of 3–5 MΩ when filled with solution containing (in mM): 90 K acetate, 20 KCl, 40 HEPES, 3 MgCl_2_, 1 CaCl_2_, 3 EGTA-KOH, 2 MgATP; pH adjusted to 7.2 with KOH. The CHO cells were continuously perfused at 1–2 ml/min with extracellular bath solution containing (in mM): 135 NaCl, 5 KCl, 5 HEPES, 1.2 MgCl_2_, 2.5 CaCl_2_, 10 glucose; pH adjusted to 7.4 by NaOH. All chemicals were purchased from Fisher Scientific (Hampton, NH) or Sigma-Aldrich. Tannic acid was added to the extracellular bath solution at concentrations between 1 and 10 μM. CHO cells were held at –80 mV, and 1 s voltage pulses were applied between from –80 to +40 mV in 20 mV increments, each followed by a 400 ms tail pulse to –30 mV, using a CV –7A Headstage (Axon Instruments, Forster City, CA). Currents were sampled at 10 kHz and filtered at 5 kHz *via* a Bessel low-pass filter. Channel voltage dependence was evaluated by fitting the tail current activation curves to a Boltzmann equation.

### Formalin Paw Lick Assay

Adult, male C57BL/6 mice (Charles River, Wilmington, MA) were group housed under a 12-h light:dark cycle and allowed access to food and water *ad libitum*. Mice were tested in the formalin paw lick assay between 9 and 12 weeks of age. The mouse study was performed under an approved Institutional Animal Care and Use Committee protocol at the University of California, Irvine.

The plant extracts prepared as described above were diluted in sterile saline and titrated to pH 7.4. The KCNQ antagonist XE991 dihydrochloride (Alomone Labs, Jerusalem, Israel) was prepared in sterile saline. The dilutions used for *in vivo* testing were estimated from the concentration required to modulate electrophysiological responses in KCNQ2/3 channels expressed in CHO cells. Experimental solutions were prepared by combining neutral buffered formalin (Sigma-Aldrich, St. Louis, MO) with the diluted plant extract, resulting in a final solution containing 2.5% formalin and 1:250 plant extract. The solution for the antagonist experiments contained 2.5% formalin, 1:250 plant extract and 10 μM XE991. The vehicle control solution was prepared by combining formalin with sterile saline, resulting in a final solution with only 2.5% formalin. All solutions were prepared fresh daily. Morphine sulfate (Spectrum Chemical, Gardena, CA) was used as a positive control and was dissolved in sterile saline at 0.5 mg/ml. Morphine was administered subcutaneously 15 min prior to formalin with an injection volume of 10 ml/kg (5 mg/kg).

Mice were habituated to the procedure room in their home cages for at least one hour prior to testing. Mice that received the 5 mg/kg morphine treatment were dosed subcutaneously 15 min prior to formalin injection. Either 2.5% formalin alone, 10 μM XE991 alone, 2.5% formalin with 10 μM XE991, 2.5% formalin with 1:250 plant extract, or 2.5% formalin with 1:250 plant extract and 10 μM XE991 was injected into the dorsal surface of the left hindpaw and then the animal was immediately placed in a large, clear polymethylpentene beaker for observation. The amount of time spent licking the injected paw was recorded in 5-min bins over 50–60 min by individuals blinded to the treatment received.

### Chemical Structures and *silico* Docking

For *in silico* ligand docking predictions of tannic and gallic acid binding to KCNQ2/3 we performed unguided docking using SwissDock with CHARMM forcefields (Grosdidier et al., 2011a,b) and a chimeric human neuronal KCNQ/*Xenopus* KCNQ1 cryo-electron microscopy model as previously described (Manville et al., 2018).

### Statistical Analysis

All data are presented as mean +SEM; all *p* values were two-sided. One-way ANOVA was applied for electrophysiological analyses. For the paw lick assay: the early phase of the formalin paw lick was defined by the first 5-min time bin. The late phase of the formalin paw lick was defined by the 10–50- or 10–60-min time bins. The statistical significance of the early and late phase average bar figures was determined by one-way analysis of variance (ANOVA) with *post hoc* analyses using Dunnett’s or Bonferroni’s multiple comparison test as appropriate. The statistical significance of the time course figures was determined by two-way ANOVA with *post hoc* analyses using Dunnett’s or Bonferroni’s multiple comparison test as appropriate.

## Results

### Functional Screening Against KCNQ2/3 of Californian Coastal Redwood Forest Plants

We collected aerial part samples (predominantly leaves) from Californian coastal redwood forest plants in Muir Woods National Monument (Figure 1B) then conducted methanol extraction (80% methanol/20% water) followed by evaporation of methanol to leave aqueous solutions. We tested effects of extracts from the first 40 plants collected, on human KCNQ2/3 channel activity using manual two-electrode voltage clamp (TEVC) electrophysiology in the *X. laevis* oocyte expression system, with 1:50 dilutions in bath solution of each extract. We screened both for shifts in resting membrane potential (*E*_M_) in unclamped, KCNQ2/3-expressing oocytes (Figure 1C) and the fold-change induced by the extracts in tail currents at –30 mV following a prepulse elicited by depolarization from –80 to –60 mV (Figure 1D).

As KCNQ2/3-dependent cell membrane hyperpolarization is expected to be the most important property for small molecules to KCNQ2/3-dependently reduce cellular excitability, we selected for further analysis the 9 plant extracts most effective at KCNQ2/3-dependent cellular hyperpolarization - *Urtica dioica* (Common Nettle), *Gaultheria shallon* (Salal), *Arctostaphylos glandulosa* (Eastwood Manzanita), *Polystichum munitum* (Western Sword Fern), *Sisyrinchium bellum* (Western Blue-Eyed Grass), *Rosa gymnocarpa* (Dwarf Rose), *Arbutus menziesii* (Pacific Madrone), *Heracleum maximum* (Common Cow Parsnip), and *Hedera canariensis* (Algerian Ivy). All hyperpolarized the membrane potential by at least –3 mV and comprised 7 of the top 10 extracts with respect to increasing KCNQ2/3 activity at –60 mV. In contrast, three extracts had no hyperpolarizing effect and instead induced mild to moderate depolarization of the cell membrane potential (by at least +2.5 mV) and were also studied further – *Berberis nervosa* (Oregon Grape), *Polygala californica* (California Milkwort), and *Elymus californicus* (California Bottlebrush Grass) (Figures 1C,D and Supplementary Table 1).

### KCNQ2/3-Dependent Cellular Hyperpolarization Correlates With Traditional Use of Plant Extracts as Analgesics and Gastrointestinal Aids

All 9 of the plant species, extracts of which induced KCNQ2/3-dependent cell membrane hyperpolarization, are documented to have been traditionally used for analgesia and related indications, and also as gastrointestinal aids, by indigenous populations in North America (Balls, 1962; Moerman, 2009), or in the case of the non-native species, *H. canariensis* (until recently classified as a *Hedera helix* subspecies) by European populations (Grieve, 1971; Chiej, 1984; Bown, 1995). Note that *U. dioica* subspecies present in California (*U. dioica* ssp. *gracilis* and *holoriceia*) first arrived in the Pleistocene, thus we treat *U. dioica* here similarly to the other native plants. *U. dioica* ssp. *dioica* is not officially represented in California; it was, however, introduced to other parts of North America by European settlers in the 1800s and entered into widespread medicinal use by Native Americans after that (Baker et al., 1991; Borchers et al., 2000; Preston and Woodland, 2012; Santucci and Tweet, 2020).

**FIGURE 2:**
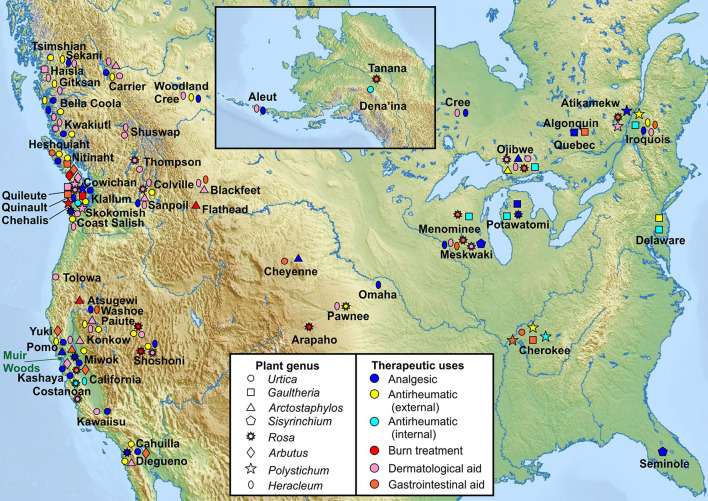
Traditional medicinal usage of KCNQ2/3-activating plants. Geographical and tribal traditional medicinal usage of 8 native plant extract “hits” from KCNQ2/3 screening, categorized by plant genus as reported in Moerman (2009) to permit comparison of indigenous medicinal use of closely related plant species native to geographically distinct regions. Approximate locations of Native American tribes are indicated by plant usage symbols and tribe names. Note that many of the tribal lands constitute much larger areas than depicted by single labels or symbols. Muir Woods location indicated by green rectangle. Upper inset: Alaska and neighboring region (Tribal location sources in Supplementary Material).

We analyzed North American indigenous tribal use of all plants in the genus of each of the 8 KCNQ2/3-activating native species and the geographical location of the tribes, to permit comparison of historical medicinal use of genetically related plant species by geographically distinct populations. The analysis indicates that culturally and geographically distinct indigenous populations demonstrated, in many cases, similar uses of the plants in the genus of the KCNQ2/3 activators as analgesics, internal and external antirheumatics, dermatologic aids (including treatment of sores, rashes, and stings by insects or other plants), and burns (Figure 2; Balls, 1962; Moerman, 2009). In contrast, none of the three KCNQ2/3 inhibitors were reportedly used for these indications (Moerman, 2009). Furthermore, none of the eight KCNQ2/3-activating species used medicinally by Native Americans were in traditional use for two indications unrelated to KCNQ2/3 activity (classified as disinfectant and liver aid) (Moerman, 2009). Because individual species are often not widely geographically represented, the plant genus analysis permitted us to compare medicinal use of relatively closely related plants living in different habitats and in locations sufficiently far apart that use may have arisen independently in some locations. From this we deduced that independently discovered efficacy was at least partly a driver for medicinal use of the plants studied, versus solely tradition driving such use. This supported the hypothesis that the plants possessed efficacy in ameliorating the conditions for which they were used and validated the pursuit of molecular mechanistic analyses. A species-specific analysis showed similar patterns of medicinal use (Supplementary Table 1).

### Traditional Analgesics Increase KCNQ2/3 Activity at Hyperpolarized Potentials

**FIGURE 3:**
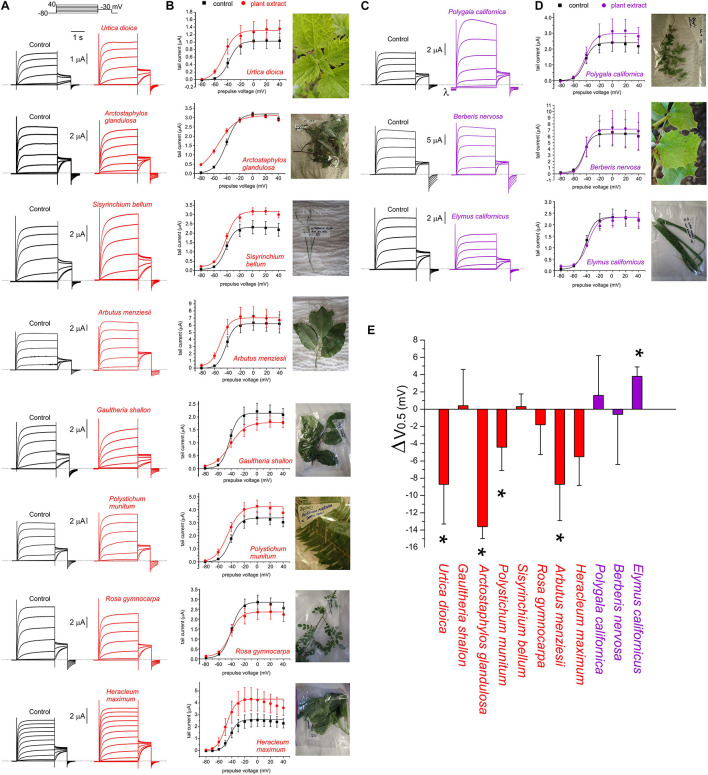
Effects of Muir Woods plants on KCNQ2/3 channel voltage dependence. All error bars indicate SEM. *n* = number of oocytes. **(A)** Exemplar TEVC current traces for KCNQ2/3-expressing *Xenopus* oocytes in the absence (Control) or presence of 1:50 (in bath solution) plant extracts found to activate KCNQ2/3 at hyperpolarized potentials (*n* = 4–11). *Upper inset*, the voltage protocol used here and throughout the study (used with either 10 or 20 mV prepulse increments) unless otherwise indicated. **(B)** Mean tail current versus prepulse voltage, for traces recorded as in panel **(A)**, for plant extracts found to activate KCNQ2/3 at hyperpolarized potentials (*n* = 5–11). **(C)** Exemplar TEVC current traces for KCNQ2/3-expressing *Xenopus* oocytes in the absence (Control) or presence of 1:50 (in bath solution) plant extracts found to not affect or to inhibit KCNQ2/3 currents at hyperpolarized potentials (*n* = 3–4). Dashed line here and throughout indicates the zero-current level. λ indicates leak induced by *P. californica*. **(D)** Mean tail current versus prepulse voltage, for traces recorded as in panel **(A)**, for plant extracts KCNQ2/3 (*n* = 3–4). **(E)** Mean shift in midpoint voltage dependence of KCNQ2/3 activation (ΔV_0.5_) calculated from plots in panels **(B)** and **(D)**. * *P* < 0.05; others, *P* > 0.05 (*n* = 3–11).

Extracts from the plants traditionally used by Native Americans as analgesics, antirheumatics, or skin treatments for burns, stings and sores, augmented KCNQ2/3 channel activity. This was often observed as both a shift in the midpoint voltage dependence (V_0.5_) of activation to more negative potentials and an increase in current at hyperpolarized membrane potentials, e.g., for *U. dioica*, *A. glandulosa*, *A. menziesii* (Figures 1D, 3A,B,E and Supplementary Table 1). In other cases, an increase in current at hyperpolarized potentials was not accompanied by a shift in V_0.5_ of activation, because the slope of the latter changed, becoming shallower (e.g., *G. shallon*) (Figures 3B,E). In contrast, three of the Muir Woods plant aerial parts extracts not historically used for analgesics, antirheumatics, or skin treatments for burns, stings and sores (*P. californica*, *B. nervosa*, and *E. californicus*) mildly to moderately depolarized cell membrane potential, and either positive-shifted or did not alter KCNQ2/3 activation V_0.5_ (Figures 1D, 3C–E and Supplementary Table 1). Application of *P. californica* induced a leak component visible at the larger negative potentials (labeled lambda in Figure 3C) and positive potentials studied, the reversal potential of which was not consistent with a K^+^ current. The tail current, measured at –30 mV, showed no change at hyperpolarized voltages compared to control and we concluded that *P. californica* was relatively KCNQ2/3 inactive, and induced non-specific leak. In sum, traditional analgesic and related uses correlated well with ability to activate KCNQ2/3 channels at hyperpolarized potentials.

### Traditional Analgesics KCNQ-Dependently Ameliorate Pain in Mice

**FIGURE 4:**
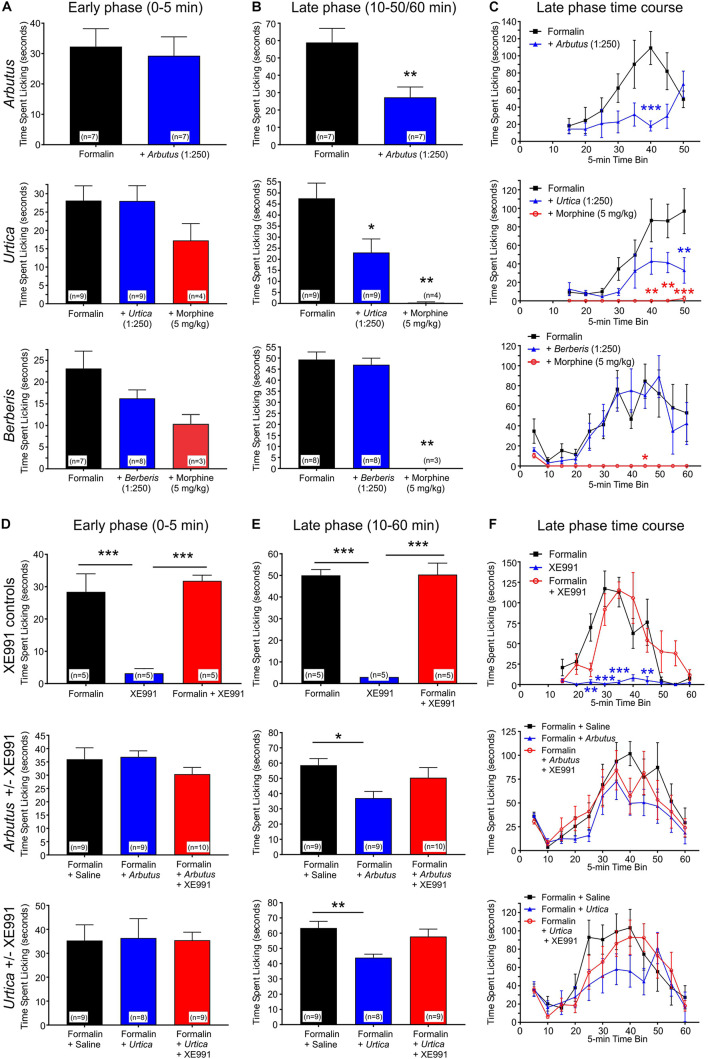
Local administration of plant extracts in a mouse formalin paw lick model. All error bars indicate SEM. *n* = number of mice. Statistical analyses for all panels were by one-way ANOVA, except where indicated as two-way ANOVA. *p < 0.05; **p < 0.01; ***p < 0.001. **(A)** Licking in the early (acute) phase of the formalin assay in the presence or absence of *A. menziesii*, *U. dioica, B. nervosa*, or morphine as indicated. **(B)** Average licking per 5-min time bin in the late (chronic) phase of the formalin assay. *Arbutus* (*p* < 0.01, *n* = 7), *Urtica* (*p* < 0.05, *n* = 9), and morphine (*p* < 0.01, *n* = 3–4) each significantly reduced paw licking in the late phase, while there was no difference with *Berberis* (*p* > 0.05, *n* = 8). **(C)** Time course by 5-min time bins for the formalin assay. In a two-way ANOVA comparing the late phase of formalin alone to formalin with plant extract, there was a significant main effect of treatment for *Arbutus* (*p* = 0.0087) and *Urtica* (*p* = 0.017), but not for *Berberis* (*p* = 0.61). **(D)** Licking in the early (acute) phase of the formalin assay. XE991 alone did not influence paw-licking, neither did it alter the effects of formalin. Similarly, neither of the plant extracts changed the amount of paw licking in the early phase regardless of the presence or absence of XE991. **(E)** Average licking per 5-min time bin in the late (chronic) phase of the formalin assay. XE991 alone did not influence paw-licking, neither did it alter the effects of formalin. In contrast, *Arbutus* (*p* < 0.05, *n* = 9) and *Urtica* (*p* < 0.01, *n* = 8) each reduced paw licking in the late phase. This effect was blocked by XE991 with both *Arbutus* and *Urtica*. **(F)** Time course by 5-min time bins for the formalin assay. In a two-way ANOVA comparing the late phase of formalin alone to formalin with plant extract, there was a statistically significant main effect of treatment for *Arbutus* (*p* = 0.0029) and *Urtica* (*p* = 0.0017), which in each case was inhibited by XE991. In contrast, XE991 had no effect alone and did not alter effects of formalin.

The effects were next determined of local administration of plant extracts in the mouse formalin paw lick assay, in which pain/irritation is quantified by the frequency of paw licking following an injection into the paw of formalin, alone or with test materials or controls. Paw licking was quantified during early (0–5 min) and late (10–50/60 min) phases post-injection. The early phase is considered a model of acute pain in which the licking is due to the pain of the needle penetration and the formalin solution entering the subcutaneous space. The reaction to this type of acute pain subsides quickly. The late phase (the period of which greatly depends on the strain) is a model of inflammatory pain involving edema, heat and pain in the affected area (Shibata et al., 1989). We tested two plant extracts, *A. menziesii* and *U. dioica*, which increased KCNQ2/3 activity at hyperpolarized potentials and one plant extract, *B. nervosa*, which did not. None of the extracts affected paw licking during the early phase. Correlating with their effect on KCNQ2/3 activation voltage dependence, and indicative of an analgesic effect, *A. menziesii* and *U. dioica* extracts each reduced paw licking in the late phase; in contrast, *B. nervosa* extract did not change the amount of paw licking (Figures 4A–C). Importantly, the analgesic effects of *A. menziesii*, and *U. dioica* extracts were KCNQ-dependent, as they were inhibited by co-injection of the relatively KCNQ-specific antagonist, XE991 (10 μM) (Figures 4D–F). In contrast, XE991 did not cause irritation when administered alone, nor did it alter effects of formalin in the absence of plant extracts (Figures 4D–F), while morphine was an effective analgesic positive control (Figures 4A–C).

### KCNQ2/3-Activating Compounds Provide a Plausible Molecular Basis for Analgesic Plant Effects

**FIGURE 5:**
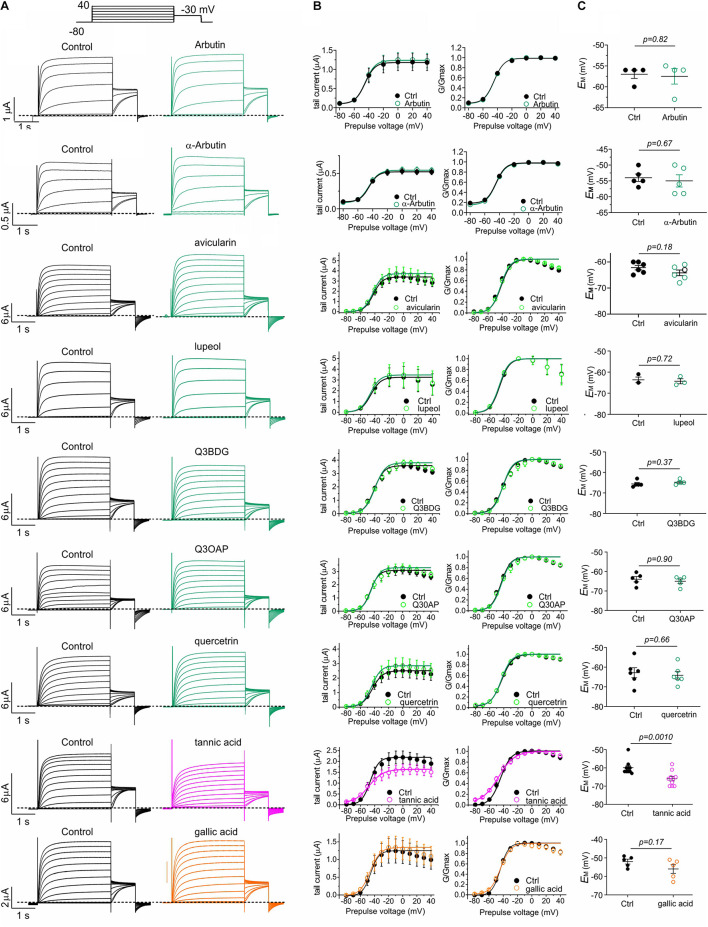
Tannic and gallic acid but not other Muir Woods plant compounds tested activate KCNQ2/3. All error bars indicate SEM. *n* = number of oocytes. **(A)** Exemplar TEVC traces of oocyte-expressed KCNQ2/3 in the absence (Control) and presence of 100 μM concentrations of compounds in Muir Woods plant “hits” (*n* = 3–10). Upper inset: voltage protocol. Q3BDG, quercetin 3-β-D-glucoside; Q3OAP, quercetin 3-O-α-L-arabinopyranoside. **(B)** Mean tail current and normalized tail currents (G/Gmax) versus prepulse voltage relationships for the traces as in panel **(A)**. **(C)** Scatter plot of unclamped membrane potential (*E*_M_) for *Xenopus* oocytes expressing KCNQ2/3. Statistical analysis by two-way ANOVA.

The five plants most effective at negative-shifting KCNQ2/3 activation V_0.5_ were *A. menziesii*, *A. glandulosa*, *U. dioica, P. munitum*, and *H. maximum* (Figure 3D). Prior analyses of *A. menziesii* and other *Arbutus* species leaf extracts (Kabadi and Hammarlund, 1963; Kouki and Manetas, 2002; Zitouni et al., 2020a,b) revealed tannic acid as a principal component, together with compounds related to quercetin, which we previously found to activate KCNQ2/3 (Redford and Abbott, 2020) (quercetrin, quercetin 3-β-D-glucoside and quercetin 3-O-α-L-arabinopyranoside), gallic acid, and avicularin. In addition, the glycosylated hydroquinone arbutin has been identified as the major abundant bioactive component in leaves of closely related *Arbutus unedo* (Kabadi and Hammarlund, 1963; Tenuta et al., 2019). Tannic acid and gallic acid are also abundant in *Arctastaphylos* (Panusa et al., 2015) and *Polystichum* species (Edwards and Vavasseur, 1831). *U. dioica* contains tannins, gallic acid, quercetin, quercetrin, quercetin 3-β-D-glucoside, and quercetin 3-O-α-L-arabinopyranoside, while lupeol is its most abundant sterol (Kregiel et al., 2018). We quantified the effects of the above compounds and also α-arbutin (each at 100 μM) on KCNQ2/3 activity and influence on *E*_M_, in *Xenopus* oocytes. Tannic acid increased KCNQ2/3 current at hyperpolarized potentials, and gallic acid to a lesser extent, while the other compounds had negligible effects (Figures 5A,B). At 100 μM, tannic acid negative-shifted *E*_M_ of KCNQ2/3-expressing oocytes by ≥-5 mV, while the other compounds had no statistically significant effects (Figure 5C). The results recapitulated previously reported effects of tannic acid on KCNQ2/3 expressed in HEK cells and on native M-current in nociceptive neurons (Zhang et al., 2015).

**FIGURE 6:**
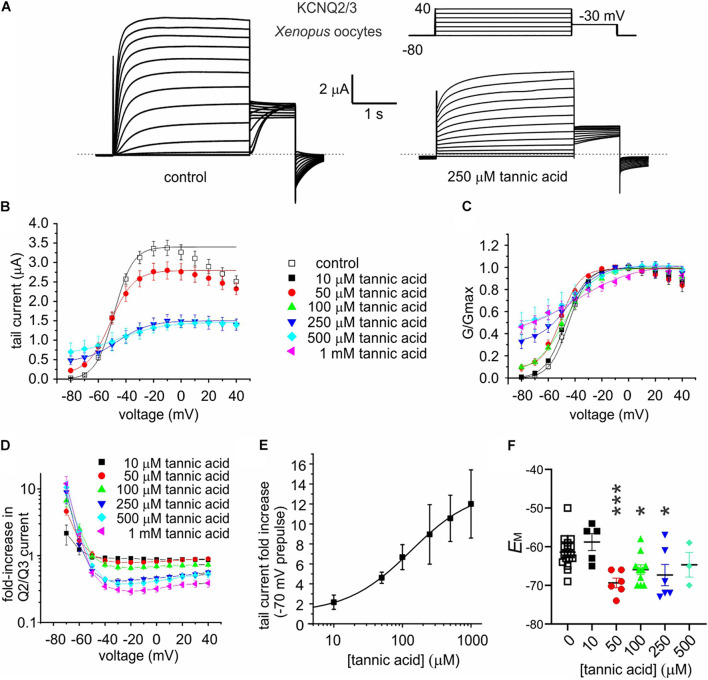
Tannic acid exerts dual effects on KCNQ2/3 channels expressed in oocytes. All error bars indicate SEM. *n* = number of oocytes. **(A)** Exemplar TEVC traces of oocyte-expressed KCNQ2/3 in the absence (control) and presence of tannic acid (250 μM). Upper right inset: voltage protocol. **(B)** Mean tail current versus prepulse voltage relationships for traces as in panel **(A)** at various tannic acid concentrations (*n*: control, 17; 10 μM, 5; 50 μM, 6; 100 μM, 10; 250 μM, 6; 500 μM, 3; 1 mM, 6). **(C)** Mean normalized tail currents (G/Gmax) versus prepulse voltage relationships for traces as in panel **(A)** at various tannic acid concentrations (*n* as in panel **B**). **(D)** KCNQ2/3 current fold-change versus voltage at various tannic acid concentrations for oocytes recorded as in panel **(A)** (*n* as in panel **B**). **(E)** Tannic acid dose response for KCNQ2/3 current increase at –70 mV for oocytes as in panel **(D)** (*n* as in panel **B**). **(F)** Scatter plot of unclamped membrane potential (*E*_M_) for cells as in panel **(A)** at various tannic acid concentrations (*n* as in panel **B**). Statistical analysis by one-way ANOVA. *p < 0.05; ***p < 0.001.

**FIGURE 7:**
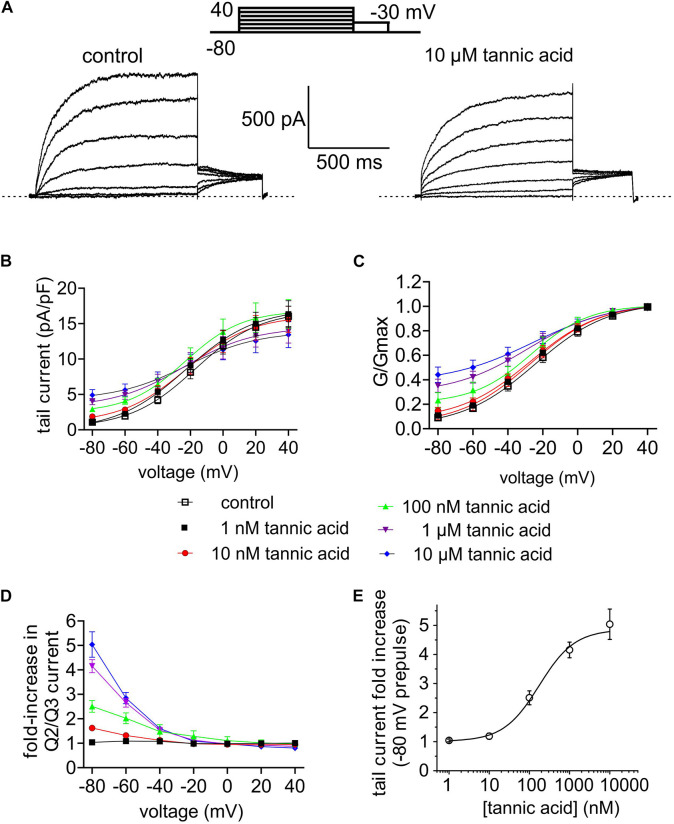
Tannic acid is a potent activator of KCNQ2/3 channels expressed in CHO cells. All error bars indicate SEM. *n* = number of cells. **(A)** Exemplar whole-cell patch clamp traces of CHO cell-expressed KCNQ2/3 in the absence (control) and presence of tannic acid (10 μM). Upper inset: voltage protocol. **(B)** Mean tail current versus prepulse voltage relationships for traces as in panel **(A)** at tannic acid concentrations indicated in key below (*n* = 10). **(C)** Mean normalized tail currents (G/Gmax) versus prepulse voltage relationships for traces as in panel **(A)** at tannic acid concentrations indicated in key below (*n* = 10). **(D)** KCNQ2/3 current fold-change versus voltage at various tannic acid concentrations indicated in key above for cells recorded as in panel **(A)** (*n* = 10). **(E)** Tannic acid dose response for KCNQ2/3 current increase at –80 mV for cells as in panel **(D)** (*n* = 10).

Tannic acid (50 μM) induced a constitutively active component at –80 mV in the KCNQ2/3 current expressed in oocytes, which was further increased at higher concentrations; concurrent with this was an inhibition at higher tannic acid concentrations of KCNQ2/3 current at more positive voltages, such that the I/V relationships at different tannic acid concentrations crossed over at around –60 mV (Figures 6A–C). At 500 μM, tannic acid increased KCNQ2/3 current tenfold at –70 mV but inhibited >twofold at –20 mV (Figure 6D). The tannic acid *EC*_50_ for KCNQ2/3 current augmentation in oocytes was 132 ± 163 μM at –70 mV (Figure 6E). Consistent with these effects, tannic acid hyperpolarized *E*_M_ in KCNQ2/3-expressing oocytes at 50 μM and higher concentrations (Figure 6F). Tannic acid slowed the time-dependent component of KCNQ2/3 activation. Fitting of the KCNQ2/3 current at –40 mV (where currents are large yet slow enough to be accurately fitted with a double exponential component) revealed that tannic acid (100 μM) slowed by >twofold both the slow (1092 ± 386 ms, tannic acid; versus 464 ± 155 ms, control; *n* = 8–9; *p* = 0.01) and the fast (244 ± 86 ms, tannic acid; versus 93 ± 31 ms, control; *n* = 8–9; *p* = 0.11) components of activation, while not altering the relative amplitudes (A) of the two components (A_fast_/(A_fast_ + A_slow_): 0.171 ± 0.060, tannic acid; versus 0.166 ± 0.055, control; *n* = 8–9; *p* = 0.97). Despite the slowing of activation, because tannic acid induces an activation component that is time-independent (constitutive) at –80 mV, the sum effect is to increase KCNQ2/3 activity at hyperpolarized membrane potentials, and thus hyperpolarize the resting membrane potential. The constitutive component is readily observable in the tannic acid-treated oocyte trace in Figure 6A (and see Figure 7A).

**FIGURE 8:**
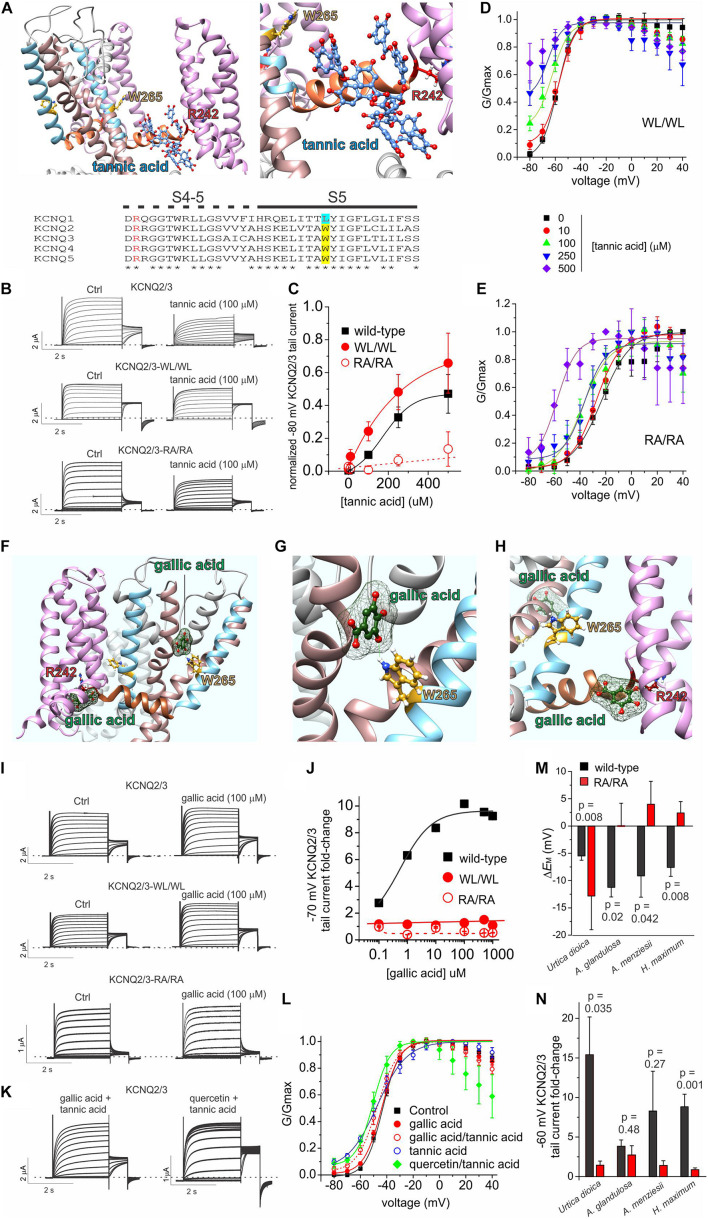
Tannic acid and gallic acid *in silico* docking and mutagenesis. All error bars indicate SEM. *n* = number of oocytes. **(A)** *In silico* docking by SwissDock showing predicted binding pose for tannic acid in KCNQ2 (*left*, far view; *right*, close-up). *Lower inset*, sequence alignment of human KCNQ channel isoforms in the S4-5 linker and S5 regions. Alignment colors: Red, KCNQ3-R242 and equivalents; yellow, KCNQ3-W265 and its equivalents in KCNQ2, 4 and 5; Cyan, KCNQ1-L266. **(B)** Exemplar TEVC traces of oocyte-expressed wild-type and mutant KCNQ2/3 in the absence (control) and presence of tannic acid (100 μM). **(C)** Mean tail current after a –80 mV prepulse, normalized to maximal tail current, for wild-type and mutant KCNQ2/3 channels as in panel **(B)**. Wild-type, *n* and source data as in Figure 6B; WL/WL, *n* = 5–6; RA/RA, *n* = 5–8, except 500 μM: *n* = 4. **(D)** Mean normalized tail currents (G/Gmax) versus prepulse voltage relationships for WL/WL traces as in panel **(B)** at various tannic acid concentrations (*n* as in panel **C**). **(E)** Mean normalized tail currents (G/Gmax) versus prepulse voltage relationships for RA/RA traces as in panel **(B)** at various tannic acid concentrations (*n* as in panel **C**). **(F)** *In silico* docking by SwissDock showing predicted binding poses for gallic acid in KCNQ3. **(G)** *In silico* docking by SwissDock showing predicted binding pose for gallic acid in KCNQ3 close to W265. **(H)** *In silico* docking by SwissDock showing predicted binding pose for gallic acid in KCNQ3 close to R242. **(I)** Exemplar TEVC traces of oocyte-expressed wild-type and mutant KCNQ2/3 in the absence (control) and presence of gallic acid (100 μM). **(J)** Fold-change in wild-type and mutant KCNQ2/3 tail current (after a –70 mV prepulse) at various gallic acid concentrations for oocytes recorded as in panel **I**. Wild-type: *n* = 6; WL/WL, *n* = 5–7; RA/RA, *n* = 6–8, except 250, 500 μM: *n* = 4–5. **(K)** Exemplar TEVC traces of oocyte-expressed wild-type KCNQ2/3 in the presence of tannic acid (100 μM) with either gallic acid or quercetin (100 μM). **(L)** Mean normalized tail currents (G/Gmax) versus prepulse voltage relationships for wild-type KCNQ2/3 in the presence of various gallic acid, tannic acid and/or quercetin combinations as indicated (*n* = 12–13 except: gallic acid, *n* = 6; tannic acid, *n* = 8). **(M)** Comparison of effects of the plant extracts named (1:50 dilution) on the resting membrane potential of oocytes expressing wild-type (black) versus RA/RA mutant (red) KCNQ2/3 channels (*n* = 4–11). Wild-type data is from Figure 1D. **(N)** Comparison of effects of the plant extracts named (1:50 dilution) on the tail current following a –60 mV prepulse recorded in oocytes expressing wild-type (black) versus RA/RA mutant (red) KCNQ2/3 channels (*n* = 4–11). Wild-type data is from Figure 1D.

The binding site for tannic acid in KCNQ channels was not previously reported. We therefore performed unbiased *in silico* docking, which predicted that tannic acid binds close to an arginine at the foot of S4, at the junction with the S4-5 linker (R213 in KCNQ2; R242 in KCNQ3) at the opposite end of the retigabine/GABA binding pocket from the S5 tryptophan residue that is essential for retigabine binding (W236 in KCNQ2; W265 in KCNQ3) (Figure 8A). To test this prediction, we compared the effects of tannic acid on wild-type KCNQ2/3 channels to effects on KCNQ2/3 with the S4-5 linker arginine mutated in both KCNQ isoforms (KCNQ2/3-RA/RA) and also on KCNQ2/3 channels with the S5 tryptophan mutated in both isoforms (KCNQ2/3-WL/WL) (Figure 8B). Comparing the ability of tannic acid to induce constitutive activation at –80 mV, a hallmark of its effects on KCNQ2/3 (Figure 6), we found that KCNQ2/3-WL/WL tannic acid sensitivity was similar to that of wild-type KCNQ2/3. In contrast, tannic acid was much less effective at inducing constitutive current at –80 mV in KCNQ2/3-RA/RA channels (Figure 8C). These findings were also reflected in normalized G/V curves for either mutant, although from these it was evident that tannic acid was still able to negatively shift the midpoint voltage dependence of KCNQ2/3-RA/RA activation (by –34.6 mV at 500 μM tannic acid, from –23.3 ± 1.5 mV at baseline to –57.9 ± 4.6 mV) (Figures 8D,E). The data are consistent with the docking prediction that tannic acid binds close to KCNQ2/3-R213/R242 and that this arginine residue is important for the ability of tannic acid to hold KCNQ2/3 open at hyperpolarized potentials, yet not essential for physical binding of tannic acid.

Gallic acid had much weaker effects than tannic acid at 100 μM (Figure 5) but we studied it further, as in previous studies of plants we discovered summative or synergistic effects of their constitutive compounds on specific channels (Manville and Abbott, 2018; Redford and Abbott, 2020). *In silico* docking predicted that in contrast to tannic acid, the much smaller gallic acid (170 Da, versus 1700 Da for tannic acid) is capable of adopting different binding positions, one close to KCNQ2/3-R213/R242 and one close to KCNQ2/3 S5 tryptophan W236/W265 (with which gallic acid is predicted to hydrogen bond) (Figures 8F–H). Gallic acid dose response studies examining fold-increase in tail current after a –70 mV prepulse, which was the most prominent effect of 100 μM gallic acid in the compound screen (Figure 5) generated two interesting findings. First, the *EC*_50_ for gallic acid augmentation of KCNQ2/3 current was 560 ± 3 nM at –70 mV (Figures 8I,J), 235-fold more potent than tannic acid under similar conditions (Figure 6E). Second, KCNQ2/3-WL/WL and KCNQ2/3 RA/RA channels were insensitive to gallic acid, even up to 1 mM (Figures 8I,J), consistent with the docking predictions of Figures 8F–H.

As gallic acid and tannic acid are both present in *Arbutus sp.* and other plants identified here to be KCNQ2/3-activating, we tested the effects of both gallic and tannic acids in combination (each at 100 μM). The effects were intermediate between those of each acid alone (Figures 8K,L). Taken together with the functional data above, these data are consistent with gallic acid being a relatively high-affinity partial agonist that interferes with tannic acid activation of KCNQ2/3 by competing for a similar binding pocket.

We previously found that quercetin negative-shifts the midpoint voltage-dependence of KCNQ1 and KCNQ2/3 activation (Redford and Abbott, 2020). Prior KCNQ1 docking and KCNQ1 and KCNQ2/3 mutagenesis data were consistent with quercetin binding sites on the VSD and F340 on S6, rather than in the retigabine/GABA binding pocket (Redford and Abbott, 2020). As some KCNQ2/3-activating plants identified herein contain both quercetin and tannic acid, we applied a combination of both (each at 100 μM) and found that rather than competing, quercetin augmented the KCNQ2/3-opening effect of tannic acid, permitting the tannic-acid-induced constitutive activation at –80 mV while also steepening the G/V curve compared to tannic acid alone (Figures 8K,L). These data, like the docking predictions, are consistent with distinct and non-competing binding locations for quercetin and tannic acid.

We next tested whether the tannic acid binding site mutations (KCNQ2/3-RA/RA) affected efficacy of the plant extracts. Compared to wild-type KCNQ2/3 channels, KCNQ2/3-RA/RA channels were much less responsive to three of the most efficacious plant extracts (*A. glandulosa, A. menziesii, H. maximum*) with respect to ability to hyperpolarize *E*_M_ (*p* < 0.05) (Figure 8M). In contrast, *U. dioica* had a stronger membrane hyperpolarizing effect on cells expressing KCNQ2/3-RA/RA channels than on those expressing wild-type KCNQ2/3 (*p* = 0.008) (Figure 8M). KCNQ2/3-RA/RA channels were less responsive to all four of the above plant extracts versus wild-type KCNQ2/3 with respect to increased activity at –60 mV, with two of these comparisons reaching a *p* value < 0.05 (*U. dioica* and *H. maximum*) (Figure 8N). The apparently paradoxical effects of *U. dioica* (greater effect on *E*_M_ in KCNQ2/3-RA/RA channels than on wild-type KCNQ2/3 but the reverse with respect to current increase at –60 mV) may be explained by the fact that *U. dioica* contains quercetin, which we previously found to act at sites on the S6 and the top of the voltage sensor, rather than at the S4-5 arginine (Redford and Abbott, 2020). Quercetin negative-shifts the voltage dependence of KCNQ2/3 activation but does not induce a constitutive current at –80 mV (Redford and Abbott, 2020), in contrast to tannic acid. Therefore, with the site required for tannic acid effects removed, it is possible that effects of quercetin were uncovered. The data are consistent with tannic acid, gallic acid and/or other compounds that require the S4-5 linker arginine, and quercetin, contributing to the KCNQ2/3-activating effects of the plant extracts tested.

Many compounds exhibit higher potency in mammalian cells than in *Xenopus* oocytes, and tannic acid was no exception. Whole-cell patch-clamp electrophysiology studies showed qualitatively similar augmentation by tannic acid of constitutive current at –80 mV and inhibition at more positive voltages, for human KCNQ2/3 expressed in CHO cells (Figures 7A–D), yet at much lower doses than for oocyte studies. Hence, the tannic acid *EC*_50_ for KCNQ2/3 current augmentation in CHO cells was 175 ± 83 nM at –80 mV (*n* = 10) (Figure 7E).

### Tannic Acid Exerts KCNE-Dependent Effects on KCNQ1 Channel Activity

**FIGURE 9:**
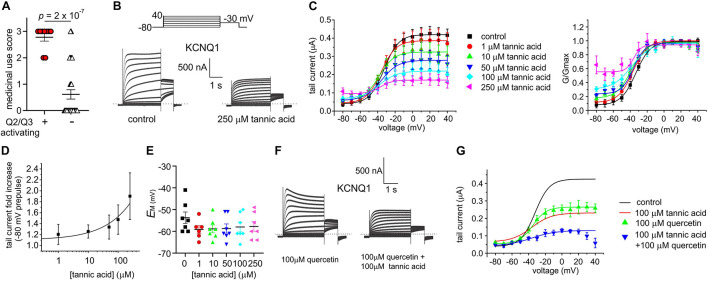
Tannic acid exerts dual effects on KCNQ1. All error bars indicate SEM. *n* = number of oocytes. **(A)** Medicinal use score (see Supplementary Table 1) for KCNQ2/3 activating versus non-activating plant extracts studied herein. **(B)** Exemplar TEVC traces of oocyte-expressed KCNQ1 in the absence (control) and presence of tannic acid (250 μM). **(C)** Mean tail current (left) and normalized tail current (G/Gmax; right) versus prepulse voltage relationships for traces as in panel **(A)** at various tannic acid concentrations (*n* = 8). **(D)** Tannic acid dose response for KCNQ1 current increase at –80 mV for oocytes as in panel **(B)** (*n* = 8). **(E)** Scatter plot of unclamped membrane potential (*E*_M_) for KCNQ1-expressing oocytes as in panel **(B)** at various tannic acid concentrations (*n* = 6–7). Statistical analysis by one-way ANOVA indicated no statistically significant differences between groups. **(F)** Exemplar TEVC traces of oocyte-expressed KCNQ1 in the presence of 100 μM quercetin alone or with tannic acid (100 μM) (*n* = 5). **(G)** Mean tail current versus prepulse voltage relationships for traces as in panel **(F)** at various tannic acid concentrations (*n* = 5); best fit lines from panel **(C)** shown for comparison.

The eight plant extracts used by Native Americans as traditional analgesics that we found to open KCNQ2/3 channels are also documented as being used as gastrointestinal aids by Native North American populations, in geographically and culturally distinct populations that suggest independent discovery and use in some cases by different tribal groups (Moerman, 2009; Figure 2). We scored each of the 40 extracts based on reported traditional medicinal use in each of 3 categories: pain/rheumatism, dermatological/burns, and gastrointestinal aids (Supplementary Table 1). Strikingly, comparing the eight KCNQ2/3-activating extracts with the 32 non-KCNQ2/3-activating extracts, a score of 3 (i.e., use in 3 of the categories) was almost exclusively predictive of KCNQ2/3 activation ability (6/8 activators versus 1/32 non-activators) while a score of 0-1 (i.e., use in 0–1 of the categories) was exclusively linked to inability to activate KCNQ2/3 (0/8 activators versus 25/32 non-activators). KCNQ2/3 activators had a mean score of 2.8 ± 0.1, while non-activators had a mean score of 0.6 ± 0.2 (*n* = 40; *p* = 2.0 × 10^–7^) (Figure 9A).

In the same gene subfamily as KCNQ2 and KCNQ3 but with very different roles and tissue localization, KCNQ1 is predominantly expressed in gastric and endocrine epithelial tissues, as well as the heart and inner ear (Abbott, 2014). KCNQ1 sensitivity to tannic acid was not previously reported. Here, we found that tannic acid constitutively opened KCNQ1 at –80 mV and inhibited it at more positive potentials (Figures 9B–D), leading to modest tannic acid-induced hyperpolarization of *E*_M_ in oocytes expressing KCNQ1, although this did not reach statistical significance (Figure 9E).

We previously found that quercetin enhances both activation and inactivation of homomeric KCNQ1 channels, inducing visible voltage-dependent decay at depolarized potentials (Redford and Abbott, 2020), recapitulated here (Figure 9F). We applied a combination of tannic acid and quercetin to KCNQ1 and found that the combination was not as effective as tannic acid alone in inducing constitutive current, but the current at less hyperpolarized potentials was inhibited more effectively than for either compound alone. In addition, tannic acid prevented speeding of inactivation by quercetin (Figures 9F,G).

**FIGURE 10:**
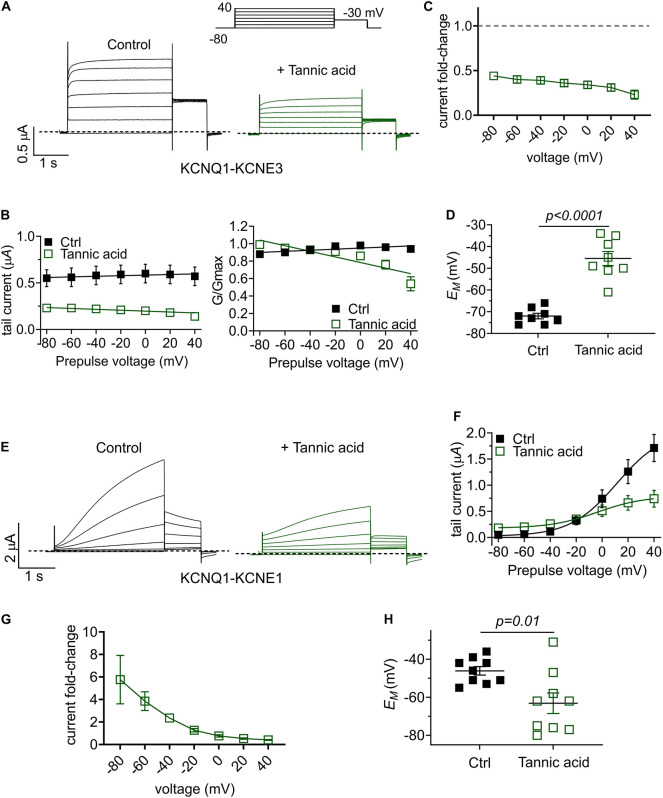
Tannic acid exerts KCNE-specific effects on KCNQ1 channel complexes. **(A)** Exemplar TEVC traces of oocyte-expressed KCNQ-KCNE3 channels in the absence (control) and presence of tannic acid (100 μM) (*n* = 8). **(B)** Mean tail current (left) and normalized tail current (G/Gmax; right) versus prepulse voltage relationships for traces as in panel **(A)** (*n* = 8). **(C)** Tannic acid-induced tail current fold-change versus prepluse voltage for oocytes as in panel **(A)**. **(D)** Scatter plot of unclamped membrane potential (*E*_M_) for KCNQ1-KCNE3-expressing oocytes as in panel **(A)** in the absence (Ctrl) or presence of tannic acid (100 μM). Statistical analysis by one-way ANOVA. **(E)** Exemplar TEVC traces of oocyte-expressed KCNQ-KCNE1 channels in the absence (control) and presence of tannic acid (100 μM) (*n* = 9). **(F)** Mean tail current versus prepulse voltage relationships for traces as in panel **(E)** (*n* = 9). **(G)** Tannic acid-induced tail current fold-change versus prepluse voltage for oocytes as in panel **(E)**. **(H)** Scatter plot of unclamped membrane potential (*E*_M_) for KCNQ1-KCNE1-expressing oocytes as in panel **(E)** in the absence (Ctrl) or presence of tannic acid (100 μM). Statistical analysis by two-way ANOVA.

One gastrointestinal therapeutic application of tannic acid is as an anti-diarrheal agent, for instance in piglets immediately after weaning (Yu et al., 2020). In the intestine, KCNQ1 co-assembles with the KCNE3 single transmembrane segment ancillary subunit, creating a heteromeric, constitutively active potassium channel that regulates chloride secretion (Schroeder et al., 2000). Inhibition of KCNQ1-KCNE3 by another compound was previously demonstrated to be a mechanism for treating diarrhea (Bajwa et al., 2007). Strikingly, we found that tannic acid (100 μM) inhibited KCNQ1-KCNE3 current by 55–75% across all voltages (Figures 10A–C), causing a mean +27 mV depolarization of oocytes expressing KCNQ1-KCNE3 (Figure 10D).

Finally, we tested the effects of tannic acid on KCNQ1-KCNE1 complexes, which are expressed in human heart and inner ear. Tannic acid (100 μM) increased KCNQ1-KCNE1 current sixfold at –80 mV, inducing >20% constitutive activation for this otherwise relatively positive-activating channel (Figures 10E–G). Despite 50% inhibition of KCNQ1-KCNE1 peak current by tannic acid at +40 mV, there was a clear “locking open” effect at all voltages tested, likely resulting from cumulative activation in successive voltage pulses, such that at +40 mV, 50% of the prepulse KCNQ1-KCNE1 current was instantaneous (Figure 10E), in contrast to the normally slow-activating channel properties in the absence of tannic acid. This resulted in a >-15 mV hyperpolarization of *E*_M_ of oocytes expressing KCNQ1-KCNE1 (Figure 10H).

## Discussion

Native North Americans used more than 3000 plant species for medicinal purposes (Moerman, 2009). Geographically distinct tribes often used the same plant genus, and in many cases the same species of plant, for similar and very specific therapeutic purposes (Figure 2). This suggests that tribes that may not have been in communication with one another independently discovered that certain plants were effective in the treatment of specific disorders. It should, however, be noted that there were thriving trade centers among different Native American tribal nations, whose members would travel in some cases hundreds of miles to barter their locally sourced goods for those of others. The Shoshoni, for instance, were very active traders, dealing with geographically distinct tribes from both the North American northwest and southwest. Bartered items included animal skins, meat, obsidian and quartzite for construction of sharp-edged tools and weapons, plant products including pumpkin, tobacco, corn, and also medicinal herbs. Thus, in addition to locally sourced medicinal plants, tribes may have had access to herbs from geographically distinct locations, and presumably knowledge of the indications for which they should be used, *via* trading. As few of the tribes developed written forms of communication, details of therapeutic uses of plants, such as species, optimal extraction, and plant combinations, were predominantly passed down the generations through oral tradition. Botanical medicine expertise and traditions could therefore be lost when practitioners perished.

Nevertheless, many records of traditional medicine use of plants have remained, albeit precise details are often lacking (Moerman, 2009). We found a striking preponderance among the plant extracts that activated KCNQ2/3, of historical use as primarily topical analgesics and treatments for burns, sores, insect bites and stings (Figure 2). Many were historically prepared as poultices for the skin or, e.g., for Cow Parsnip, as a topical analgesic for toothache. KCNQ channels, including KCNQ2/3 heteromers, are expressed in peripheral nociceptive pathways and their activation can reduce responsiveness to stimuli (Passmore et al., 2003; Passmore et al., 2012). The antinociceptive effects of *Arbutus andrachne L.* and *U. dioica* extracts following systemic administration have been previously reported (Dhouibi et al., 2018; Jaffal et al., 2020), but we report the first demonstration of the antinociceptive efficacy of *A. menziesii* and *U. dioica* administered locally, better recapitulating their use primarily as topical analgesics (often on broken skin such as sores, wounds and burns) by Native Americans. Furthermore, we also demonstrate for the first time that their action is KCNQ-dependent and provide evidence for several active components within the plants that underlie effects on KCNQ channel activity.

Our data demonstrate that KCNQ channel activation is a prominent underlying mechanism of action of at least two of the traditional plant remedies (*A. menziesii* and *U. dioica*) we found to activate KCNQ2/3 channels *in vitro*. Consistent with use of tannic-acid containing, KCNQ2/3-activating plants as topical analgesics, others previously found that tannic acid is effective as a topical agent for improvement burn wound healing and reducing burn pain, and for osteoarthritis (Halkes et al., 2001; Smith and Jacobson, 2011). In addition, prior studies on tannic acid activation of KCNQ2/3 suggested particular utility for tannic acid in treating bradykinin-associated inflammatory pain, which might result from burns but also arthritis and rheumatism (Zhang et al., 2015), both indications for which Native Americans utilized the KCNQ2/3-activating, tannic acid-containing plants described herein.

All the plants we found to activate KCNQ2/3 were used as gastrointestinal aids, in addition to their use as analgesics. Use of the same plant species for two such apparently disparate indications is herein explained by the ubiquity and diversity of the various KCNQ channels isoforms and their pharmacological flexibility bestowed by KCNE regulatory subunits (Anantharam et al., 2003; Panaghie and Abbott, 2006; Wrobel et al., 2016). One common specific gastrointestinal indication of the plants identified in the KCNQ2/3 activation screen was to ameliorate diarrhea (Moerman, 2009). Interestingly, tannic acid is effective in ameliorating diarrhea in piglets post-weaning (Yu et al., 2020). Furthermore, inhibition of intestinal basolateral KCNQ1-KCNE3 channels using fenofibrate was previously found to inhibit cAMP- or cGMP-stimulated Cl^–^ secretion (which is regulated by KCNQ1-KCNE3) in response to, e.g., heat-stable enterotoxin, and suggested as a safe, effective antidiarrheal drug (Bajwa et al., 2007). Given these findings, we investigated additional effects of tannic acid - which is present in several of the plant extracts we discovered to activate KCNQ2/3 - and found that it inhibits KCNQ1-KCNE3 across the voltage range. This effect is KCNE3-dependent as tannic acid effects on both KCNQ1 and KCNQ1-KCNE1 were more similar to those on KCNQ2/3, i.e., augmenting at hyperpolarized potentials. The versatility of tannic acid in augmenting KCNQ2/3, KCNQ1, and KCNQ1-KCNE1 activity at hyperpolarized potentials but inhibiting KCNQ1-KCNE3 mechanistically rationalizes the use by Native Americans of tannic acid-containing plants as both topical analgesics and antidiarrheals. We found a tannic acid *EC*_50_ of 175 nM for activation of KCNQ2/3 channels when expressed in mammalian cells; others previously found low-μM *EC*_50_ values using a different voltage protocol and HEK cell expression (Zhang et al., 2015) whereas we used CHO cells. Either of these potencies are well within the range that could be achieved on, e.g., an affected broken skin surface or in the intestinal epithelia, either by topical application or by ingestion, respectively, given that polyphenol levels in the plasma, for example, can reach in the hundreds of μM after consumption of polyphenol-rich foods (Manach et al., 2004). Inhibition of KCNQ1-KCNE1 (I_Ks_) channels in the heart, such as occurs in our experiments with tannic acid in oocytes, would potentially be arrhythmogenic, yet this effect has not been reported in instances for which tannic acid has been used, e.g., as an antidiarrheal drug (Yu et al., 2020). The likely explanation is that the ingestion of tannic acid at the dose needed to inhibit channels expressed in the gut epithelium would not be sufficient to produce high enough free tannic acid plasma levels to access and modulate channels in the heart; this explanation is supported by, e.g., previous findings that tannic acid binds to endogenous proteins in the intestinal lumen, probably limiting its absorption compared to, e.g., catechin (Carbonaro et al., 2001).

In summary, Californian coastal redwood forest plants are a rich source of bioactive molecules and have been used extensively as food and medicine by the indigenous peoples of what is now Marin County. Similar plants (same genus and in some cases same species) to those in the redwood forests were used across North America by many different tribes, often for analogous therapeutic indications. Our random sample in Muir Woods revealed that more than 20% of plants were able to activate KCNQ2/3 potassium channels. While tannic acid, gallic acid and/or quercetin likely underlie the action of several of the plants we identified to activate KCNQ2/3, it is feasible that other small molecules contribute that remain to be characterized. Larger-scale screens across plants from varied habitats may reveal higher-potency small molecules with KCNQ2/3-activating properties that have drug potential, given the high hit-rate from our smaller screen. However, the rush to reduce to a single drug-like active component can be counterproductive, potentially sacrificing safety, efficacy or both in favor of convenience and scalability. Indigenous Native American herbalists were undoubtably adept at their art and there is still much to learn from their techniques and approaches. The therapeutic utility and versatility of Californian native plants further highlights the absolute necessity to prevent and reverse the damage done to their habitats by climate change and other factors.

## Data Availability Statement

The raw data supporting the conclusions of this article will be made available by the authors, without undue reservation.

## Ethics Statement

The animal study was reviewed and approved by Institutional Animal Care and Use Committee, University of California, Irvine.

## Author Contributions

GWA conceived the project, helped to collect plants, screened and functionally characterized effects of plant extracts and compounds with TEVC, conducted *in silico* docking, analyzed data, wrote the manuscript, prepared the figures, and obtained funding for the project. KR, LM, and RM conducted electrophysiological analyses and/or *in silico* docking, analyzed data, and prepared figures. RY conducted pain assays, analyzed data, and prepared figures. KT performed plant extractions and analysis on tribal use of plants. GA, AK, EL, and EG coordinated field studies, collected and identified plants. All authors read and edited the manuscript.

## Conflict of Interest

The authors declare that the research was conducted in the absence of any commercial or financial relationships that could be construed as a potential conflict of interest.

## Publisher’s Note

All claims expressed in this article are solely those of the authors and do not necessarily represent those of their affiliated organizations, or those of the publisher, the editors and the reviewers. Any product that may be evaluated in this article, or claim that may be made by its manufacturer, is not guaranteed or endorsed by the publisher.

## References

[B1] Abbott G. W. (2014). Biology of the KCNQ1 potassium channel. *New J. Sci.* 2014:237431.

[B2] Abbott G. W. (2020). KCNQs: ligand- and voltage-gated potassium channels. *Front. Physiol.* 11:583. 10.3389/fphys.2020.00583 32655402 PMC7324551

[B3] Abd-Elsayed A. Jackson M. Gu S. L. Fiala K. Gu J. (2019). Neuropathic pain and Kv7 voltage-gated potassium channels: the potential role of Kv7 activators in the treatment of neuropathic pain. *Mol. Pain* 15:1744806919864256. 10.1177/1744806919864256 PMC6659175 31342847

[B4] Abd-Elsayed A. A. Ikeda R. Jia Z. Ling J. Zuo X. Li M. (2015). KCNQ channels in nociceptive cold-sensing trigeminal ganglion neurons as therapeutic targets for treating orofacial cold hyperalgesia. *Mol. Pain* 11:45. 10.1186/s12990-015-0048-8 26227020 PMC4521366

[B5] Anantharam A. Markowitz S. M. Abbott G. W. (2003). Pharmacogenetic considerations in diseases of cardiac ion channels. *J. Pharmacol. Exp. Ther.* 307 831–838. 10.1124/jpet.103.054569 14561846

[B6] Bajwa P. J. Alioua A. Lee J. W. Straus D. S. Toro L. Lytle C. (2007). Fenofibrate inhibits intestinal Cl- secretion by blocking basolateral KCNQ1 K+ channels. *Am. J. Physiol. Gastrointest. Liver Physiol.* 293 G1288–G1299. 10.1152/ajpgi.00234.2007 17916649

[B7] Baker R. G. Schwert D. P. Bettis E. A. I. Kemmis T. J. Horton D. G. Semken H. A. (1991). Mid-Wisconsinan stratigraphy and paleoenvironments at the St. Charles site in south-central Iowa. *GSA Bull.* 103 210–220. 10.1130/0016-7606(1991)103<0210:mwsapa>2.3.co;2

[B8] Balls E. K. (1962). *Early Uses of California Plants.* Los Angeles, CA: University of California Press.

[B9] Bean L. J. (1994). *The Ohlone Past and Present: Native Americans of the San Francisco Bay Region.* Novata, CA: Ballena Press.

[B10] Biervert C. Schroeder B. C. Kubisch C. Berkovic S. F. Propping P. Jentsch T. J. (1998). A potassium channel mutation in neonatal human epilepsy. *Science* 279 403–406. 9430594 10.1126/science.279.5349.403

[B11] Blackburn-Munro G. Jensen B. S. (2003). The anticonvulsant retigabine attenuates nociceptive behaviours in rat models of persistent and neuropathic pain. *Eur. J. Pharmacol.* 460 109–116. 10.1016/s0014-2999(02)02924-2 12559370

[B12] Borchers A. T. Keen C. L. Stern J. S. Gershwin M. E. (2000). Inflammation and Native American medicine: the role of botanicals. *Am. J. Clin. Nutr.* 72 339–347. 10.1093/ajcn/72.2.339 10919925

[B13] Bown D. (1995). *Encyclopedia of Herbs and Their Uses.* London: Dorling Kindersley.

[B14] Carbonaro M. Grant G. Pusztai A. (2001). Evaluation of polyphenol bioavailability in rat small intestine. *Eur. J. Nutr.* 40 84–90. 10.1007/s003940170020 11518204

[B15] Chiej R. (1984). *Encyclopedia of Medicinal Plants.* London: MacDonald.

[B16] Dhouibi R. Moalla D. Ksouda K. Ben Salem M. Hammami S. Sahnoun Z. (2018). Screening of analgesic activity of Tunisian *Urtica dioica* and analysis of its major bioactive compounds by GCMS. *Arch. Physiol. Biochem.* 124 335–343. 10.1080/13813455.2017.1402352 29157001

[B17] Du X. Gao H. Jaffe D. Zhang H. Gamper N. (2018). M-type K(+) channels in peripheral nociceptive pathways. *Br. J. Pharmacol.* 175 2158–2172. 10.1111/bph.13978 28800673 PMC5980636

[B18] Edwards H. M. Vavasseur M. D. (1831). *A Manual of Materia Medica and Pharmacy.* London: Whittaker, Treacher, and Co.

[B19] Grieve M. (1971). *A Modern Herbal: The Medicinal, Culinary, Cosmetic and Economic Properties, Cultivation and Floklore of Herbs, Grasses, Fungi, Shrubs, & Trees with All Their Modern Scientific Uses.* New York, NY: Dover Publications.

[B20] Grosdidier A. Zoete V. Michielin O. (2011a). Fast docking using the CHARMM force field with EADock DSS. *J. Comput. Chem.* 32 2149–2159. 10.1002/jcc.21797 21541955

[B21] Grosdidier A. Zoete V. Michielin O. (2011b). SwissDock, a protein-small molecule docking web service based on EADock DSS. *Nucleic Acids Res.* 39 W270–W277. 10.1093/nar/gkr366 21624888 PMC3125772

[B22] Halkes S. B. Van Den Berg A. J. Hoekstra M. J. Du Pont J. S. Kreis R. W. (2001). Treatment of burns: new perspectives for highly purified tannic acid? *Burns* 27 299–300. 10.1016/s0305-4179(00)00104-2 11383524

[B23] Hardy K. Buckley S. Collins M. J. Estalrrich A. Brothwell D. Copeland L. (2012). Neanderthal medics? Evidence for food, cooking, and medicinal plants entrapped in dental calculus. *Naturwissenschaften* 99 617–626. 10.1007/s00114-012-0942-0 22806252

[B24] Harkcom W. T. Papanikolaou M. Kanda V. Crump S. M. Abbott G. W. (2019). KCNQ1 rescues TMC1 plasma membrane expression but not mechanosensitive channel activity. *J. Cell. Physiol.* 234 13361–13369. 10.1002/jcp.28013 30613966 PMC6478532

[B25] Heizer R. F. Whipple M. A. (1971). *The California Indians: A Source Book.* Berkely, CA: University of California Press.

[B26] Inskeep R. R. (1969). Health hazards and healing in antiquity. *S. Afr. Archaeol. Bull.* 24 21–29. 10.2307/3888363

[B27] Jaffal S. M. Oran S. A. Alsalem M. (2020). Anti-nociceptive effect of *Arbutus andrachne* L. methanolic leaf extract mediated by CB1, TRPV1 and PPARs in mouse pain models. *Inflammopharmacology* 28 1567–1577. 10.1007/s10787-020-00746-y 32935246

[B28] Kabadi B. Hammarlund E. R. (1963). Preliminary identification of the antibacterial principle “Madronin” from the leaves of *Arbutus menziesii*. *J. Pharm. Sci.* 52 1154–1159. 10.1002/jps.2600521212 14088965

[B29] Klinger F. Gould G. Boehm S. Shapiro M. S. (2011). Distribution of M-channel subunits KCNQ2 and KCNQ3 in rat hippocampus. *Neuroimage* 58 761–769. 10.1016/j.neuroimage.2011.07.003 21787867 PMC3166433

[B30] Kouki M. Manetas Y. (2002). Toughness is less important than chemical composition of *Arbutus* leaves in food selection by *Poecilimon* species. *New Phytol.* 154 399–407. 10.1046/j.1469-8137.2002.00375.x 33873438

[B31] Kregiel D. Pawlikowska E. Antolak H. (2018). *Urtica* spp.: ordinary plants with extraordinary properties. *Molecules* 23:1664. 10.3390/molecules23071664 29987208 PMC6100552

[B32] Lange W. Geissendorfer J. Schenzer A. Grotzinger J. Seebohm G. Friedrich T. (2009). Refinement of the binding site and mode of action of the anticonvulsant retigabine on KCNQ K+ channels. *Mol. Pharmacol.* 75 272–280. 10.1124/mol.108.052282 19015229

[B33] Main M. J. Cryan J. E. Dupere J. R. Cox B. Clare J. J. Burbidge S. A. (2000). Modulation of KCNQ2/3 potassium channels by the novel anticonvulsant retigabine. *Mol. Pharmacol.* 58 253–262. 10.1124/mol.58.2.253 10908292

[B34] Manach C. Scalbert A. Morand C. Remesy C. Jimenez L. (2004). Polyphenols: food sources and bioavailability. *Am. J. Clin. Nutr.* 79 727–747. 10.1093/ajcn/79.5.727 15113710

[B35] Manville R. W. Abbott G. W. (2018). Ancient and modern anticonvulsants act synergistically in a KCNQ potassium channel binding pocket. *Nat. Commun.* 9:3845. 10.1038/s41467-018-06339-2 30242262 PMC6155021

[B36] Manville R. W. Abbott G. W. (2019). Cilantro leaf harbors a potent potassium channel-activating anticonvulsant. *FASEB J.* 33 11349–11363. 10.1096/fj.201900485R 31311306 PMC6766653

[B37] Manville R. W. Abbott G. W. (2020). Potassium channels act as chemosensors for solute transporters. *Commun. Biol.* 3:90. 10.1038/s42003-020-0820-9 32111967 PMC7048750

[B38] Manville R. W. Papanikolaou M. Abbott G. W. (2018). Direct neurotransmitter activation of voltage-gated potassium channels. *Nat. Commun.* 9:1847. 10.1038/s41467-018-04266-w PMC5945843 29748663

[B39] Manville R. W. Van Der Horst J. Redford K. E. Katz B. B. Jepps T. A. Abbott G. W. (2019). KCNQ5 activation is a unifying molecular mechanism shared by genetically and culturally diverse botanical hypotensive folk medicines. *Proc. Natl. Acad. Sci. U.S.A.* 116 21236–21245. 10.1073/pnas.1907511116 31570602 PMC6800379

[B40] Matschke V. Piccini I. Schubert J. Wrobel E. Lang F. Matschke J. (2016). The natural plant product Rottlerin activates Kv7.1/KCNE1 channels. *Cell. Physiol. Biochem.* 40 1549–1558. 10.1159/000453205 27997884

[B41] Moerman D. E. (2009). *Native American Medicinal Plants – An Ethnobotanical Dictionary.* Portland, OR: Timber Press.

[B42] Panaghie G. Abbott G. W. (2006). The impact of ancillary subunits on small-molecule interactions with voltage-gated potassium channels. *Curr. Pharm. Des.* 12 2285–2302. 10.2174/138161206777585175 16787255

[B43] Panusa A. Petrucci R. Marrosu G. Multari G. Gallo F. R. (2015). UHPLC-PDA-ESI-TOF/MS metabolic profiling of *Arctostaphylos pungens* and *Arctostaphylos uva-ursi*. A comparative study of phenolic compounds from leaf methanolic extracts. *Phytochemistry* 115 79–88. 10.1016/j.phytochem.2015.01.002 25702282

[B44] Passmore G. M. Reilly J. M. Thakur M. Keasberry V. N. Marsh S. J. Dickenson A. H. (2012). Functional significance of M-type potassium channels in nociceptive cutaneous sensory endings. *Front. Mol. Neurosci.* 5:63. 10.3389/fnmol.2012.00063 22593734 PMC3351001

[B45] Passmore G. M. Selyanko A. A. Mistry M. Al-Qatari M. Marsh S. J. Matthews E. A. (2003). KCNQ/M currents in sensory neurons: significance for pain therapy. *J. Neurosci.* 23 7227–7236. 10.1523/jneurosci.23-18-07227.2003 12904483 PMC6740665

[B46] Preston R. E. Woodland D. W. (2012). *Urtica dioica [Online]*. Available online at: https://ucjeps.berkeley.edu/eflora/eflora_display.php?tid=47575 (accessed October 27, 2021).

[B47] Redford K. E. Abbott G. W. (2020). The ubiquitous flavonoid quercetin is an atypical KCNQ potassium channel activator. *Commun. Biol.* 3:356. 10.1038/s42003-020-1089-8 32641720 PMC7343821

[B48] Santucci V. L. Tweet J. S. (2020). *Grand Canyon National Park Centennial Paleotological Resource Inventory in Natural Resource Report NPS/GRCA/NRR—2020/2103.* Fort Collins, CO: National Park Service.

[B49] Schenzer A. Friedrich T. Pusch M. Saftig P. Jentsch T. J. Grotzinger J. (2005). Molecular determinants of KCNQ (Kv7) K+ channel sensitivity to the anticonvulsant retigabine. *J. Neurosci.* 25 5051–5060. 10.1523/jneurosci.0128-05.2005 15901787 PMC6724866

[B50] Schroeder B. C. Waldegger S. Fehr S. Bleich M. Warth R. Greger R. (2000). A constitutively open potassium channel formed by KCNQ1 and KCNE3. *Nature* 403 196–199. 10.1038/35003200 10646604

[B51] Shibata M. Ohkubo T. Takahashi H. Inoki R. (1989). Modified formalin test: characteristic biphasic pain response. *Pain* 38 347–352. 10.1016/0304-3959(89)90222-4 2478947

[B52] Singh N. A. Charlier C. Stauffer D. Dupont B. R. Leach R. J. Melis R. (1998). A novel potassium channel gene, KCNQ2, is mutated in an inherited epilepsy of newborns. *Nat. Genet.* 18 25–29. 10.1038/ng0198-25 9425895

[B53] Smith D. B. Jacobson B. H. (2011). Effect of a blend of comfrey root extract (*Symphytum officinale* L.) and tannic acid creams in the treatment of osteoarthritis of the knee: randomized, placebo-controlled, double-blind, multiclinical trials. *J. Chiropr. Med.* 10 147–156. 10.1016/j.jcm.2011.01.003 22014903 PMC3259911

[B54] Tedeschi G. Scipioni L. Papanikolaou M. Abbott G. W. Digman M. A. (2021). Fluorescence Fluctuation Spectroscopy enables quantification of potassium channel subunit dynamics and stoichiometry. *Sci. Rep.* 11:10719. 10.1038/s41598-021-90002-2 34021177 PMC8140153

[B55] Tenuta M. C. Tundis R. Xiao J. Loizzo M. R. Dugay A. Deguin B. (2019). *Arbutus* species (Ericaceae) as source of valuable bioactive products. *Crit. Rev. Food Sci. Nutr.* 59 864–881. 10.1080/10408398.2018.1551777 30582347

[B56] Tzingounis A. V. Heidenreich M. Kharkovets T. Spitzmaul G. Jensen H. S. Nicoll R. A. (2010). The KCNQ5 potassium channel mediates a component of the afterhyperpolarization current in mouse hippocampus. *Proc. Natl. Acad. Sci. U.S.A.* 107 10232–10237. 10.1073/pnas.1004644107 20534576 PMC2890451

[B57] Wang H. S. Pan Z. Shi W. Brown B. S. Wymore R. S. Cohen I. S. (1998). KCNQ2 and KCNQ3 potassium channel subunits: molecular correlates of the M-channel. *Science* 282 1890–1893. 10.1126/science.282.5395.1890 9836639

[B58] Weyrich L. S. Duchene S. Soubrier J. Arriola L. Llamas B. Breen J. (2017). Neanderthal behaviour, diet, and disease inferred from ancient DNA in dental calculus. *Nature* 544 357–361. 10.1038/nature21674 28273061

[B59] Wickenden A. D. Yu W. Zou A. Jegla T. Wagoner P. K. (2000). Retigabine, a novel anti-convulsant, enhances activation of KCNQ2/Q3 potassium channels. *Mol. Pharmacol.* 58 591–600. 10.1124/mol.58.3.591 10953053

[B60] Wrobel E. Rothenberg I. Krisp C. Hundt F. Fraenzel B. Eckey K. (2016). KCNE1 induces fenestration in the Kv7.1/KCNE1 channel complex that allows for highly specific pharmacological targeting. *Nat. Commun.* 7:12795. 10.1038/ncomms12795 27731317 PMC5064022

[B61] Yu J. Song Y. Yu B. He J. Zheng P. Mao X. (2020). Tannic acid prevents post-weaning diarrhea by improving intestinal barrier integrity and function in weaned piglets. *J. Anim. Sci. Biotechnol.* 11:87. 10.1186/s40104-020-00496-5 32884745 PMC7460753

[B62] Zhang X. Zhang H. Zhou N. Xu J. Si M. Jia Z. (2015). Tannic acid modulates excitability of sensory neurons and nociceptive behavior and the Ionic mechanism. *Eur. J. Pharmacol.* 764 633–642. 10.1016/j.ejphar.2015.06.048 26134502

[B63] Zitouni H. Hssaini L. Ouaabou R. Viuda-Martos M. Hernandez F. Ercisli S. (2020a). Exploring antioxidant activity, organic acid, and phenolic composition in strawberry tree fruits (*Arbutus unedo* L.) growing in Morocco. *Plants* 9:1677. 10.3390/plants9121677 33266055 PMC7760662

[B64] Zitouni H. Hssaini L. Zerhoune M. Ourradi H. Viuda-Martos M. Hernandez F. (2020b). Phytochemical components and bioactivity assessment among twelve strawberry (*Arbutus unedo* L.) genotypes growing in Morocco using chemometrics. *Foods* 9:1345. 10.3390/foods9101345 32977623 PMC7598283

