# Supplementary material for: KCNQ and KCNE Isoform-Dependent Pharmacology Rationalizes Native American Dual Use of Specific Plants as Both Analgesics and Gastrointestinal Therapeutics
Source: Front Physiol. 2021 Nov 11;12:777057. doi: 10.3389/fphys.2021.777057 (PMC8632246; doi:10.3389/fphys.2021.777057)
Supplement: Supplementary file 1 [file Data_Sheet_1.PDF]

Sample #	Species	Common name	ΔE_M (mV)	current fold change	Pain/ rheum	Burn/ derm	GI	Use score
1	*Marah oregana*	Wild cucumber	-1.5±0.6	1.7±0.8	+	+	+	3
2	*Lathyria latifolius*	Everlasting pea	-0.3±0.9	1.1±0.5				0
3	*Urtica dioica*	Stinging nettle	-5.5±0.8	15±4.8	+	+	+	3
4	*Iris douglasiana*	Douglas Iris	-0.5±0.6	2.3±0.7				0
5	*Gaultheria shallon*	Salal	-7.4±1.4	3.3±1.2		+	+	2
6	*Anthoxanthum occidentale*	Sweet grass	0.5±0.6	0.4±0.1	+	+		2
7	*Iris sp.*	Iris	1.5±1.5	0.5±0.3				0
8	*Polygala californica*	California milkwort	11.8±3.4	1.1±0.6				0
9	*Calystegia occidentalis*	Western morning glory	-0.3±1.2	1.4±0.2				0
10	*Clematis vitalba*	Old Man's Beard	0.3±0.3	1.4±0.1				0
11	*Polypodium glycyrrhiza*	Licorice fern	-2.0±1.2	1.4±0.2	+		+	2
12	*Lilium pardalinum*	Tiger lily	-1.0±1.5	1.0±0.2				0
13	*Taxus brevifolia*	Pacific yew	1.5±2.5	1.0±0.5	+	+	+	3
14	*Hedera canariensis*	Algerian ivy	-5.5±3.5	1.7±0.6	+	+	+	3
15	*Arctostaphylos glandulosa*	Eastwood manzanita	-11±1.8	3.8±0.8	+	+	+	3
16	*Polystichum munitum*	Western Sword fern	-3.6±2.2	3.3±0.7	+	+	+	3
17	*Lolium perenne L. ssp*	Italian wild rye	-1.5±0.8	3.3±1.2				0
18	*Aristida pallens*	Bentgrass	-1.8±1.2	1.4±0.02				0
19	*Stipa pulchra*	Purple needle grass	-1.5±0.8	1.4±0.2				0
20	*Acacia dealbata*	Silver wattle	-1.8±0.7	1.5±0.3		+	+	2
21	*Notholithocarpus densiflorus*	Tanoak	-1.8±0.6	1.8±0.4				0
22	*Berberis nervosa*	Oregon grape	3.0±2.6	1.0±0.4	+			1
23	*Aesculus californica*	California buckeye (flowers)	-1.8±0.4	1.6±0.4				0
24	*Sisyrinchium bellum*	Western blue-eyed grass	-4.5±1.6	2.1±0.3	+		+	2
25	*Sildacea calycosa*	Checker mallow	-2.8±0.4	1.9±0.3				0
26	*Nemophila heterophylla*	Small Baby Blue Eyes	-2.5±0.3	1.9±0.4				0
27	*Sanicula crassicaulis*	Pacific black snakeroot	-0.2±2.1	1.5±0.4		+		1
28	*Anemone grayi*	Blue windflower	-1.2±0.6	1.6±0.2				0
29	*Elymus californicus*	California Bottle Brush Grass	2.8±2.7	1.3±0.5				0
30	*Rosa gymnocarpa*	Dwarf rose	-4.6±0.8	1.5±0.2	+	+	+	3
31	*Arnica discoidea*	Rayless arnica	-0.5±1.3	1.6±0.5				0
32	*Tellima grandiflora*	Fringe Cup	-2.3±1.4	2.8±1.6				0
33	*Sedum spatulifolium*	Stone Crop	0.7±1.3	1.4±0.1			+	1
34	*Euphorbia peplus*	Petty Spurge	-1.9±1.0	1.6±0.5				0
35	*Salix lasiolepis*	Arroyo willow	-1.5±1.1	2.3±0.4		+	+	2
36	*Sambucus racemose*	Red elderberry	1.6±1.4	1.2±0.1	+	+		2
37	*Gastridium pheloides*	Nit grass	0.75±1.0	0.7±0.1				0
38	*Arbutus menziesii*	Pacific Madrone	-9.1±3.9	8.3±5.0	+	+	+	3
39	*Plantago erecta*	Dotseed plantain	1.2±0.6	0.6±0.7				0
40	*Heracelum maximum*	Common cow parsnip	-7.6±1.6	8.8±1.6	+	+	+	3

Supplementary Table 1. Indigenous use of Muir Woods plant species and their effects on KCNQ2/3 channels.

Current fold change = fold change induced by 1:50 plant extract in tail current after a -60 mV prepulse. ΔE_M = shift in resting membrane potential. “Burn/derm”, used as a topical therapy for burns, sores, bites, and/or stings; “Pain/rheum”, used as an analgesic and/or rheumatism treatment. Traditional uses attributed only to roots or bark are not included as these parts were not collected/tested. Score is calculated from recorded indigenous use in each of the therapeutic categories in the table (indicated by ‘+’), primarily from (*4*) with additional literature searches to provide reasonable verification of lack of recorded use in specific categories.

Supplementary Information – sources for tribe/First Nations locations

Algonquin, Quebec
https://www.canadashistory.ca/explore/politics-law/algonquin-territory
Potawatomi https://www.mpm.edu/plan-visit/educators/wirp/history
Delaware
http://delawaretribe.org/services-and-programs/historic-preservation/removal-history-of-t
he-delaware-tribe/
Iroquois https://www.britannica.com/topic/Northeast-Indian
Menominee https://www.glitc.org/tribes-served/menominee-indian-tribe-of-wisconsin/
Ojibwe (Chippewa/Ojibwa) https://project.geo.msu.edu/geogmich/ojibwe.html
https://project.geo.msu.edu/geogmich/ojibwe.html#:~:text=The%20Chippewa%20Indian
s%2C%20also%20known,the%20Ottawa%20and%20Potawatomi%20Indians.
Tete-de-boule (Atikamekw)
https://www.cbc.ca/news/indigenous/atikamekw-first-nation-declares-sovereignty-over-it
s-territory-1.2761105
Cherokee https://www.tribalpedia.com/us-tribes/a-l/cherokee-nation/
Nitinaht https://www.nitinaht.com/first-nation/
Quinault
https://www.usgs.gov/media/images/location-quinault-indian-reservation-within-queets-q
uinault
Bella Coola (Nuxalk) https://stoningtongallery.com/tribe/nuxalk-bella-coola/

Quileute https://www7.nau.edu/itep/main/tcc/Tribes/pn_quileute
Clallam (Klallam) https://www7.nau.edu/itep/main/tcc/Tribes/pn_sklallam
Cheyenne
https://tribalnations.mt.gov/northerncheyenne#:~:text=The%20Cheyenne%20Nation%20i
s%20comprised,%2Dnative%2Fnative%20owned%20businesses.
Pomo, little lake https://factcards.califa.org/cai/pomo.html
Miwok https://factcards.califa.org/cai/miwok.html
Atsugewi https://factcards.califa.org/cai/atsugewi.html
Blackfeet https://tribalnations.mt.gov/blackfeet
Cahuilla https://factcards.califa.org/cai/cahuilla.html
Carrier http://www.canadahistoryproject.ca/1500/1500-11-dene.html
Konkow (Concow) http://www.rosevillehistorical.org/before-1820
Sanpoil https://native-land.ca/maps/territories/san-poil/
Flathead
http://www.nativepartnership.org/site/PageServer?pagename=PWNA_Native_Reservatio
ns_Flathead https://www.britannica.com/topic/Flathead-people
Meskwaki https://www.meskwaki.org/about-us/history/
Seminole https://www.semtribe.com/stof/history/introduction
https://americanindian.si.edu/nk360/removal-six-nations/seminole/map
Chehalis https://www.chehalistribe.org/our-story/people-of-the-sands/
Arapaho https://www.tribalpedia.com/us-tribes/a-l/arapaho-tribe/
Costanoan (Ohlone) https://factcards.califa.org/cai/costanoan.html
Tanana https://www.tananachiefs.org/about/communities/tanana/
Okanagn-colville https://www.colvilletribes.com/tribal-chiefs
Shoshone https://commons.marymount.edu/jowetttopic/different-tribes/
Thompson https://www.everyculture.com/North-America/Thompson.html
Paiute https://native-land.ca/maps/territories/northern-paiute/
Pawnee
https://pawneenation.org/pawnee-history/#:~:text=The%20Pawnee%20Nation%20of%20Oklahoma,North%20Platt%20River%20in%20Nebraska.
Skokomish https://www.skokomish.org/culture-and-history/
Yuki https://factcards.califa.org/cai/yuki.html
Cowichan
https://cowichantribes.com/about-cowichan-tribes/land-base/traditional-territory
Hesquiat
https://www.thecanadianencyclopedia.ca/en/article/hesquiaht#:~:text=Part%20of%20the%20Nuu%2Dchah,west%20coast%20of%20Vancouver%20Island.
Kawaiisu https://www.parks.ca.gov/?page_id=24579
Kwakiutl https://stoningtongallery.com/tribe/kwakwakawakw-kwakiutl-kwagiulth/
Diegueno https://www.britannica.com/topic/Diegueno
https://factcards.califa.org/cai/diegueno.html
Haisla https://stoningtongallery.com/tribe/haisla/
Tanaina
https://www.encyclopedia.com/humanities/encyclopedias-almanacs-transcripts-and-maps

/tanaina#:~:text=Tanaina%20are%20located%20in%20the,and%20social%20intercha
nge
%20Including%20intermarriage.
Tolowa https://www.tolowa-nsn.gov/
